# 2-(2-Pyrid­yl)pyridinium bis­(pyridine-2,6-dicarboxyl­ato-κ^3^ *O*,*N*,*O*′)aluminate(III) trihydrate

**DOI:** 10.1107/S1600536808015973

**Published:** 2008-06-07

**Authors:** Janet Soleimannejad, Hossein Aghabozorg, Yaghoub Mohammadzadeh, Shabnam Hooshmand

**Affiliations:** a Department of Chemistry, Faculty of Science, Ilam University, Ilam, Iran; b Faculty of Chemistry, Tarbiat Moallem University, 49 Mofateh Avenue, Tehran, Iran

## Abstract

The title compound, (C_10_H_9_N_2_)[Al(C_7_H_3_NO_4_)_2_]·3H_2_O or (2,2′-bipyH)[Al(pydc)_2_]·3H_2_O (where 2,2′-bipy is 2,2′-bipyridine and pydcH_2_ is pyridine-2,6-dicarboxylic acid), was synthesized by the reaction of aluminium(III) nitrate nona­hydrate with pyridine-2,6-dicarboxylic acid and 2,2′-bipyridine in a 1:2:4 molar ratio in aqueous solution. This compound is composed of an anionic complex, [Al(pydc)_2_]^−^, a protonated 2,2′-bipyridine mol­ecule as a counter-ion, (2,2′-bipyH)^+^, and three uncoordinated water mol­ecules. The anion is a six-coordinate complex, with the Al^III^ atom in a distorted octa­hedral geometry coordinated by two tridentate pyridine-2,6-dicarboxyl­ate groups. In the crystal structure, inter­molecular O—H⋯O, N—H⋯O, N—H⋯N and C—H⋯O hydrogen bonds, π–π stacking between two aromatic rings [centroid–centroid distance = 3.827 (10) Å], and C=O⋯π stacking [with distances of 3.2311 (13), 3.4924 (14) and 3.5731 (13) Å], connect the various components to form a supra­molecular structure.

## Related literature

For related literature, see: Aghabozorg *et al.* (2007 ▶, 2008 ▶); Aghabozorg, Ghadermazi & Attar Gharamaleki (2006 ▶); Aghabozorg, Ghadermazi & Ramezanipour (2006 ▶).
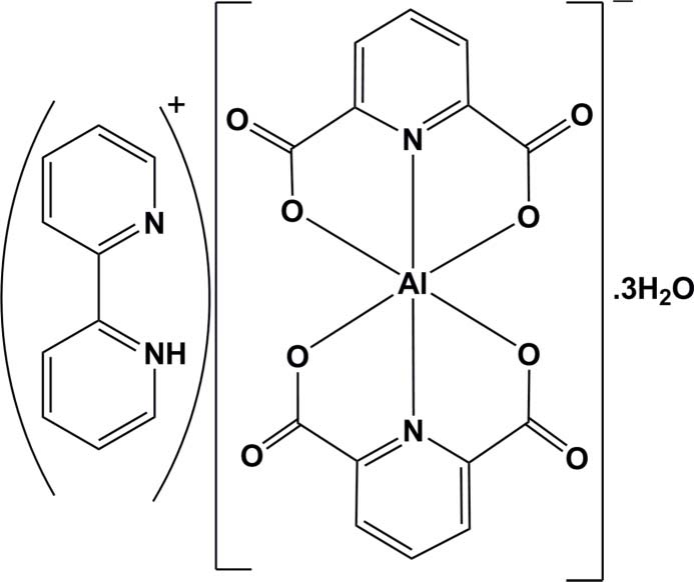


## Experimental

### 

#### Crystal data

(C_10_H_9_N_2_)[Al(C_7_H_3_NO_4_)_2_]·3H_2_O
*M* *_r_* = 568.43
Triclinic, 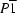

*a* = 9.3744 (13) Å
*b* = 10.9039 (16) Å
*c* = 13.005 (2) Å
α = 106.335 (7)°
β = 98.889 (7)°
γ = 97.521 (7)°
*V* = 1238.9 (3) Å^3^
*Z* = 2
Mo *K*α radiation
μ = 0.15 mm^−1^
*T* = 150 (2) K
0.32 × 0.32 × 0.15 mm

#### Data collection

Bruker SMART APEXII diffractometer
Absorption correction: multi-scan (*SADABS*; Sheldrick, 1996 ▶) *T*_min_ = 0.952, *T*_max_ = 0.977
25116 measured reflections
4350 independent reflections
3975 reflections with *I* > 2σ(*I*)
*R*_int_ = 0.028

#### Refinement

*R*[*F* ^2^ > 2σ(*F* ^2^)] = 0.029
*wR*(*F* ^2^) = 0.082
*S* = 1.07
4350 reflections
361 parameters
H-atom parameters constrained
Δρ_max_ = 0.21 e Å^−3^
Δρ_min_ = −0.27 e Å^−3^

### 

Data collection: *APEX2* (Bruker, 2007 ▶); cell refinement: *SAINT* (Bruker, 2007 ▶); data reduction: *SAINT*; program(s) used to solve structure: *SHELXS97* (Sheldrick, 2008 ▶); program(s) used to refine structure: *SHELXL97* (Sheldrick, 2008 ▶); molecular graphics: *SHELXTL* (Sheldrick, 2008 ▶); software used to prepare material for publication: *SHELXL97*.

## Supplementary Material

Crystal structure: contains datablocks I, global. DOI: 10.1107/S1600536808015973/su2057sup1.cif

Structure factors: contains datablocks I. DOI: 10.1107/S1600536808015973/su2057Isup2.hkl

Additional supplementary materials:  crystallographic information; 3D view; checkCIF report

## Figures and Tables

**Table 1:** Hydrogen-bond geometry (Å, °)

*D*—H⋯*A*	*D*—H	H⋯*A*	*D*⋯*A*	*D*—H⋯*A*
O1*S*—H1*B*⋯O2	0.85	1.98	2.8166 (14)	166
O1*S*—H1*A*⋯O3*S*^i^	0.85	1.92	2.7472 (18)	165
O2*S*—H2*A*⋯O1*S*^ii^	0.85	1.92	2.7650 (17)	175
O2*S*—H2*B*⋯O6	0.85	1.92	2.7703 (16)	174
O3*S*—H3*B*⋯O7	0.85	2.02	2.8647 (16)	170
O3*S*—H3*A*⋯O2*S*^iii^	0.85	1.94	2.7886 (18)	172
N3—H3*C*⋯O4^iv^	0.85	2.04	2.7312 (15)	138
N3—H3*C*⋯N4	0.85	2.31	2.6497 (19)	104
C12—H12⋯O1*S*^i^	0.95	2.46	3.372 (2)	160
C15—H15⋯O4^iv^	0.95	2.52	2.965 (2)	109
C16—H16⋯O2*S*^iii^	0.95	2.33	3.248 (2)	162
C17—H17⋯O1^v^	0.95	2.25	3.136 (2)	155
C18—H18⋯O8^vi^	0.95	2.50	3.331 (2)	146
C1—O1⋯*Cg*1^vii^	1.22 (1)	3.49 (1)	3.9906 (17)	105 (1)
C7—O4⋯*Cg*2^vi^	1.22 (1)	3.23 (1)	3.4319 (17)	89 (1)
C1—O1⋯*Cg*3^vii^	1.22 (1)	3.57 (1)	3.8161 (18)	92 (1)

Symmetry codes: (i) 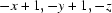; (ii) 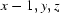; (iii) 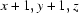; (iv) 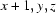; (v) 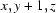; (vi) 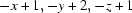. *Cg*1, *Cg*2 and *Cg*3 are the centroids of the N1/C2–C6, N3/C15–C19 and N4/C20–C24 rings, respectively.

**Fig. 1.**
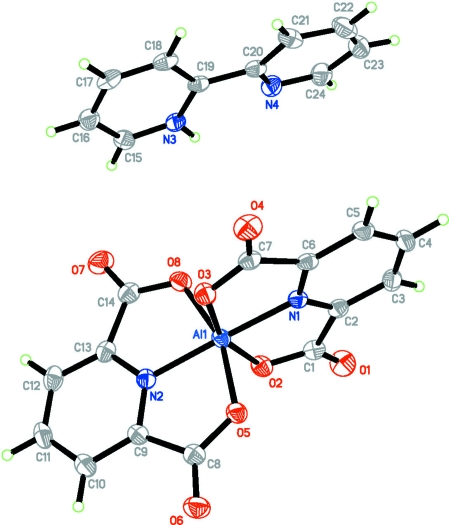
The molecular structure of the title compound, with displacement ellipsoids drawn at the 50% probability level. Uncoordinated water molecules are omitted for clarity.

**Fig. 2.**
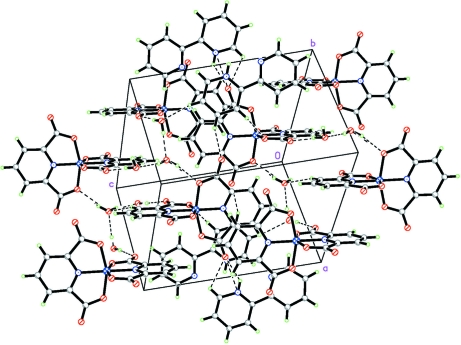
The crystal packing of the title compound with hydrogen bonds shown as dashed lines.

**Fig. 3.**
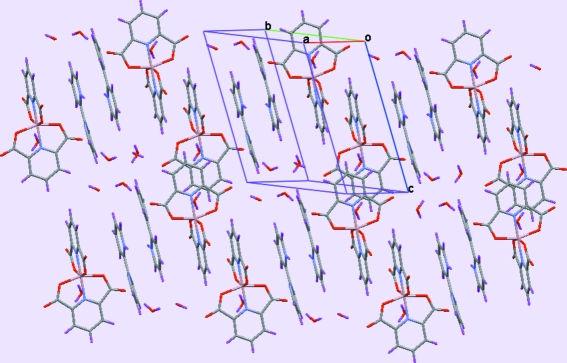
Layered diagram of the title compound. The space between the two layers of [Al(pydc)2]- fragments is filled with a layer of (2,2'-bipyH)+ cations and water molecules.

**Fig. 4.**
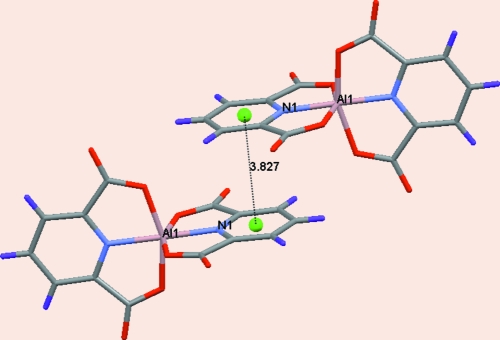
The π-π stacking between the aromatic rings of Cg1 (Cg1: N1/C2—C6) and Cg1i with distances of 3.8271 (10) Å (i = -x, 1 - y, 1 - z).

**Fig. 5.**
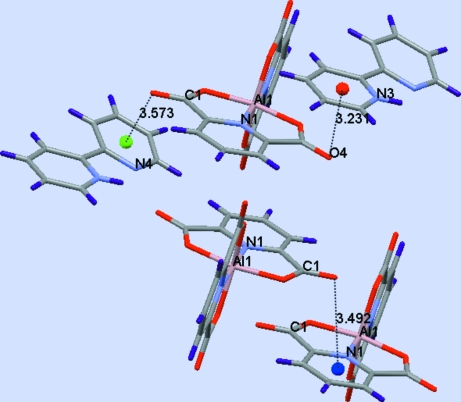
The C═O···π stacking interactions between C1═O1 and Cg1, C7═O4 and Cg2 [Cg2 centroid of ring N3/C15—C19] and C1—O1 and Cg3 [Cg3 centroid of ring N4/C20—C24] with O···π distances of 3.4924 (14) Å (1 - x, 1 - y, 1 - z), 3.2311 (13) Å (1 - x, 2 - y, 1 - z) and 3.5731 (15) Å (1 - x, 1 - y, 1 - z), respectively.

## supplementary crystallographic information

### Comment

Our research interests are centered on the preparation of water soluble proton transfer compounds as novel self assembled systems that can function as suitable ligands in the synthesis of metal complexes. In this regard, we have reported cases in which proton transfer from pyridine-2,6-dicarboxylic acid, pydcH_2_, and benzene-1,2,4,5-tetracarboxylicacid, btcH_4_, to propane-1,3-diamine (pn) and 1,10-phenanthroline, (phen), has occured. This work has resulted in the formation of some novel proton transfer compounds such as (pnH_2_)(pydc).(pydcH_2_).2.5H_2_O (Aghabozorg, Ghadermazi, Ramezanipour, 2006), (pnH_2_)_2_(btc).2H_2_O (Aghabozorg, *et al.*, 2007) and (phenH)_4_(btcH_3_)_2_(btcH_2_) (Aghabozorg, Ghadermazi, Attar Gharamaleki, 2006). For more details and related literature see our recent review article (Aghabozorg, *et al.*, 2008).

The molecular structure and the crystal packing diagram of the title compound, (2,2'-bipyH)[Al(pydc)_2_].3H_2_O, are shown in Figs. 1 and 2, respectively. The title compound is composed of an anionic complex, [Al(pydc)_2_]^-^, protonated 2,2'-bipyridine as a counter ion, (2,2'-bipyH)^+^, and three uncoordinated water molecules. The Al^III^ atom is six-coordinated by two pyridine-2,6-dicarboxylate, (pydc)^2-^, groups which act as a tridentate ligand through two O and one N atoms. The O5—Al1—O3 and O8—Al1—O2 angles (90.72 (5)° and 91.91 (5)°, repectively) and O5—Al1—O2—C1 and O5—Al1—O3—C7 torsion angles (-98.48 (10)° and 97.57 (10)°, respectively) show that these two (pydc)^2-^ anions are almost perpendicular to one another. So the anionic complex has a distorted octahedral geometry around the Al^III^ atom. For balancing the anionic complex, a protonated 2,2'-bipyridinium cation, (2,2'-bipyH)^+^, is present. The O2—Al1—O3 [159.56 (5)^°^] and O5—Al1—O8 [159.66 (5)^°^] bond angles indicate that the four carboxylate groups of the two dianions are oriented in a flattened tetrahedral arrangement around the Al^III^ atom.

In the crystal structure of the title compound, the spaces between two layers of [Al(pydc)_2_]^-^ anions are filled with (2,2'-bipyH)^+^ cations and water molecules (Fig. 3). An important feature of the title compound is the presence of π-π and C═O···π staking interactions. The π-π stacking between the aromatic rings of *Cg*1 (*Cg*1: N1/C2—C6) and *Cg*1 [-*x*, 1 - *y*, 1 - *z*], with distances of 3.8271 (10) Å , are observed in Fig. 4. The C═O···π stacking interactions between C1═ O1 and *Cg*1, C7═O4 and *Cg*2 [*Cg*2 centroid of ring N3/C15—C19] and C1═O1 and *Cg*3 [*Cg*3 centroid of ring N4/C20—C24] with O···π distances of 3.4924 (14) Å (1 - *x*, 1 - *y*, 1 - *z*), 3.2311 (13) Å (1 - *x*, 2 - *y*, 1 - *z*) and 3.5731 (15) Å (1 - *x*, 1 - *y*, 1 - *z*), respectively, are shown in Fig. 5. Intermolecular O—H···O, N—H···O, N—H···N and C—H···O hydrogen bonds, D···A ranging from 2.6497 (19) Å to 3.372 (2) Å (Table 1), appear to be effective in the stabilization of the crystal structure, resulting in the formation of an interesting supramolecular structure.

### Experimental

A solution of Al(NO_3_)_3_.9H_2_O (187 mg, 0.5 mmol) in water (5 ml) was added to an aqueous solution of pyridine-2,6-dicarboxylic acid (167 mg, 1 mmol) and 2,2'-bipyridine (312 mg, 2 mmol) in water (10 ml) in a 1:2:4 molar ratio and refluxed for an hour. Colourless crystals of the title compound were obtained after allowing the mixture to stand for two months at room temperature

### Refinement

The H-atoms were included in calculated positions and treated as riding atoms: O—H = 0.85 Å and C—H = 0.95 Å with U_iso_(H) = 1.2U_eq_(parent O or C-atom).

### Crystal data

**Table:**

(C_10_H_9_N_2_)[Al(C_7_H_3_NO_4_)_2_]·3H_2_O	*Z* = 2
*M**_r_* = 568.43	*F*_000_ = 588
Triclinic, *P*1	*D*_x_ = 1.524 Mg m^−^^3^
*a* = 9.3744 (13) Å	Mo *K*α radiation λ = 0.71073 Å
*b* = 10.9039 (16) Å	Cell parameters from 14815 reflections
*c* = 13.005 (2) Å	θ = 2.2–30.5º
α = 106.335 (7)º	µ = 0.15 mm^−^^1^
β = 98.889 (7)º	*T* = 150 (2) K
γ = 97.521 (7)º	Block, colourless
*V* = 1238.9 (3) Å^3^	0.32 × 0.32 × 0.15 mm

### Data collection

**Table:**

Bruker SMART APEXII diffractometer	4350 independent reflections
Radiation source: fine-focus sealed tube	3975 reflections with *I* > 2σ(*I*)
Monochromator: graphite	*R*_int_ = 0.028
Detector resolution: 100 pixels mm^-1^	θ_max_ = 25.0º
*T* = 150(2) K	θ_min_ = 1.7º
ω scans	*h* = −11→11
Absorption correction: multi-scan(SADABS; Sheldrick, 1996)	*k* = −12→12
*T*_min_ = 0.952, *T*_max_ = 0.977	*l* = −15→15
25116 measured reflections	

### Refinement

**Table:**

Refinement on *F*^2^	Secondary atom site location: difference Fourier map
Least-squares matrix: full	Hydrogen site location: inferred from neighbouring sites
*R*[*F*^2^ > 2σ(*F*^2^)] = 0.029	H-atom parameters constrained
*wR*(*F*^2^) = 0.082	*w* = 1/[σ^2^(*F*_o_^2^) + (0.0387*P*)^2^ + 0.5394*P*] where *P* = (*F*_o_^2^ + 2*F*_c_^2^)/3
*S* = 1.07	(Δ/σ)_max_ < 0.001
4350 reflections	Δρ_max_ = 0.21 e Å^−^^3^
361 parameters	Δρ_min_ = −0.26 e Å^−^^3^
Primary atom site location: structure-invariant direct methods	Extinction correction: none

### Special details

**Table:**

Geometry. All e.s.d.'s (except the e.s.d. in the dihedral angle between two l.s. planes) are estimated using the full covariance matrix. The cell e.s.d.'s are taken into account individually in the estimation of e.s.d.'s in distances, angles and torsion angles; correlations between e.s.d.'s in cell parameters are only used when they are defined by crystal symmetry. An approximate (isotropic) treatment of cell e.s.d.'s is used for estimating e.s.d.'s involving l.s. planes.
Refinement. Refinement of *F*^2^ against ALL reflections. The weighted *R*-factor *wR* and goodness of fit *S* are based on *F*^2^, conventional *R*-factors *R* are based on *F*, with *F* set to zero for negative *F*^2^. The threshold expression of *F*^2^ > σ(*F*^2^) is used only for calculating *R*-factors(gt) *etc*. and is not relevant to the choice of reflections for refinement. *R*-factors based on *F*^2^ are statistically about twice as large as those based on *F*, and *R*- factors based on ALL data will be even larger.

### Fractional atomic coordinates and isotropic or equivalent isotropic displacement parameters (Å^2^)

**Table:**

	*x*	*y*	*z*	*U*_iso_*/*U*_eq_	
Al1	0.21091 (4)	0.51034 (4)	0.27510 (3)	0.01853 (11)	
O1S	0.48869 (13)	0.28901 (11)	0.14144 (10)	0.0373 (3)	
H1B	0.4435	0.3080	0.1940	0.045*	
H1A	0.4252	0.2450	0.0845	0.045*	
O1	0.42412 (12)	0.30145 (11)	0.43024 (9)	0.0342 (3)	
O2S	−0.31795 (13)	0.11693 (11)	0.13006 (10)	0.0375 (3)	
H2A	−0.3805	0.1670	0.1356	0.045*	
H2B	−0.2550	0.1602	0.1069	0.045*	
O2	0.32880 (11)	0.38761 (10)	0.30284 (8)	0.0236 (2)	
O3S	0.67386 (15)	0.84922 (12)	0.06284 (10)	0.0452 (3)	
H3B	0.6320	0.8194	0.1066	0.054*	
H3A	0.6690	0.9293	0.0864	0.054*	
O3	0.09123 (10)	0.64189 (9)	0.30110 (8)	0.0223 (2)	
O4	−0.01691 (11)	0.76366 (10)	0.42483 (9)	0.0288 (2)	
O5	0.03877 (10)	0.37739 (9)	0.21149 (8)	0.0219 (2)	
O6	−0.10199 (11)	0.24420 (10)	0.05363 (9)	0.0308 (3)	
O7	0.52641 (12)	0.71867 (11)	0.19033 (10)	0.0353 (3)	
O8	0.38105 (11)	0.63460 (10)	0.28478 (8)	0.0247 (2)	
N1	0.20788 (12)	0.53539 (11)	0.42795 (9)	0.0176 (2)	
N2	0.21186 (12)	0.48075 (11)	0.12106 (9)	0.0196 (2)	
N3	0.80638 (13)	0.94087 (11)	0.48351 (10)	0.0218 (3)	
H3C	0.8551	0.8850	0.4983	0.026*	
N4	0.88910 (14)	0.93496 (12)	0.68605 (11)	0.0281 (3)	
C1	0.35290 (15)	0.37505 (14)	0.40033 (12)	0.0225 (3)	
C2	0.28187 (14)	0.46620 (13)	0.47934 (12)	0.0198 (3)	
C3	0.28675 (16)	0.48476 (14)	0.58962 (12)	0.0242 (3)	
H3	0.3400	0.4366	0.6273	0.029*	
C4	0.21100 (16)	0.57643 (15)	0.64353 (12)	0.0256 (3)	
H4	0.2134	0.5918	0.7195	0.031*	
C5	0.13182 (15)	0.64574 (14)	0.58781 (12)	0.0230 (3)	
H5	0.0785	0.7070	0.6242	0.028*	
C6	0.13321 (14)	0.62270 (13)	0.47794 (11)	0.0186 (3)	
C7	0.06099 (14)	0.68351 (13)	0.39694 (12)	0.0200 (3)	
C8	0.00324 (15)	0.32954 (13)	0.10632 (11)	0.0214 (3)	
C9	0.10685 (15)	0.39025 (13)	0.04805 (11)	0.0204 (3)	
C10	0.10349 (17)	0.36498 (15)	−0.06256 (12)	0.0256 (3)	
H10	0.0298	0.3001	−0.1154	0.031*	
C11	0.21247 (17)	0.43838 (15)	−0.09363 (12)	0.0283 (3)	
H11	0.2128	0.4234	−0.1692	0.034*	
C12	0.32087 (17)	0.53326 (15)	−0.01609 (13)	0.0270 (3)	
H12	0.3945	0.5836	−0.0375	0.032*	
C13	0.31778 (15)	0.55176 (13)	0.09321 (12)	0.0218 (3)	
C14	0.41978 (16)	0.64420 (14)	0.19568 (12)	0.0242 (3)	
C15	0.79042 (16)	0.94582 (14)	0.38086 (12)	0.0263 (3)	
H15	0.8373	0.8930	0.3301	0.032*	
C16	0.70599 (16)	1.02761 (14)	0.34927 (13)	0.0275 (3)	
H16	0.6944	1.0329	0.2769	0.033*	
C17	0.63797 (15)	1.10235 (14)	0.42492 (13)	0.0263 (3)	
H17	0.5782	1.1587	0.4040	0.032*	
C18	0.65647 (15)	1.09555 (14)	0.53085 (13)	0.0242 (3)	
H18	0.6096	1.1470	0.5825	0.029*	
C19	0.74391 (15)	1.01321 (13)	0.56095 (12)	0.0212 (3)	
C20	0.77715 (15)	0.99738 (13)	0.67040 (12)	0.0235 (3)	
C21	0.69590 (17)	1.04216 (15)	0.74973 (13)	0.0291 (3)	
H21	0.6190	1.0878	0.7361	0.035*	
C22	0.72998 (19)	1.01853 (17)	0.84872 (13)	0.0365 (4)	
H22	0.6753	1.0461	0.9040	0.044*	
C23	0.8444 (2)	0.95436 (17)	0.86625 (14)	0.0374 (4)	
H23	0.8698	0.9369	0.9337	0.045*	
C24	0.92146 (19)	0.91581 (16)	0.78373 (14)	0.0340 (4)	
H24	1.0016	0.8735	0.7971	0.041*	

### Atomic displacement parameters (Å^2^)

**Table:**

	*U*^11^	*U*^22^	*U*^33^	*U*^12^	*U*^13^	*U*^23^
Al1	0.0203 (2)	0.0193 (2)	0.0161 (2)	0.00520 (16)	0.00554 (16)	0.00375 (17)
O1S	0.0395 (6)	0.0426 (7)	0.0333 (6)	0.0104 (5)	0.0203 (5)	0.0089 (5)
O1	0.0352 (6)	0.0344 (6)	0.0376 (7)	0.0191 (5)	0.0059 (5)	0.0131 (5)
O2S	0.0413 (7)	0.0376 (7)	0.0444 (7)	0.0122 (5)	0.0182 (5)	0.0221 (6)
O2	0.0253 (5)	0.0244 (5)	0.0221 (5)	0.0103 (4)	0.0078 (4)	0.0043 (4)
O3S	0.0632 (8)	0.0363 (7)	0.0417 (7)	0.0082 (6)	0.0206 (6)	0.0160 (6)
O3	0.0250 (5)	0.0227 (5)	0.0208 (5)	0.0084 (4)	0.0056 (4)	0.0067 (4)
O4	0.0281 (5)	0.0250 (6)	0.0371 (6)	0.0137 (5)	0.0120 (5)	0.0085 (5)
O5	0.0235 (5)	0.0227 (5)	0.0188 (5)	0.0032 (4)	0.0070 (4)	0.0042 (4)
O6	0.0276 (6)	0.0302 (6)	0.0273 (6)	−0.0036 (5)	0.0019 (5)	0.0034 (5)
O7	0.0324 (6)	0.0314 (6)	0.0403 (7)	−0.0042 (5)	0.0137 (5)	0.0091 (5)
O8	0.0252 (5)	0.0244 (5)	0.0216 (5)	0.0021 (4)	0.0065 (4)	0.0028 (4)
N1	0.0165 (5)	0.0168 (6)	0.0182 (6)	0.0022 (4)	0.0041 (4)	0.0035 (5)
N2	0.0218 (6)	0.0195 (6)	0.0191 (6)	0.0070 (5)	0.0066 (5)	0.0059 (5)
N3	0.0227 (6)	0.0185 (6)	0.0256 (6)	0.0079 (5)	0.0051 (5)	0.0069 (5)
N4	0.0301 (7)	0.0266 (7)	0.0295 (7)	0.0076 (5)	0.0061 (5)	0.0104 (6)
C1	0.0193 (7)	0.0210 (7)	0.0263 (8)	0.0049 (6)	0.0032 (6)	0.0059 (6)
C2	0.0166 (6)	0.0185 (7)	0.0232 (7)	0.0011 (5)	0.0023 (5)	0.0064 (6)
C3	0.0237 (7)	0.0262 (8)	0.0229 (7)	0.0011 (6)	0.0028 (6)	0.0105 (6)
C4	0.0285 (8)	0.0278 (8)	0.0181 (7)	−0.0017 (6)	0.0063 (6)	0.0055 (6)
C5	0.0236 (7)	0.0197 (7)	0.0233 (8)	0.0000 (6)	0.0103 (6)	0.0015 (6)
C6	0.0163 (6)	0.0150 (7)	0.0223 (7)	0.0002 (5)	0.0064 (5)	0.0022 (6)
C7	0.0172 (6)	0.0164 (7)	0.0253 (8)	0.0018 (5)	0.0057 (6)	0.0044 (6)
C8	0.0221 (7)	0.0202 (7)	0.0219 (8)	0.0077 (6)	0.0049 (6)	0.0046 (6)
C9	0.0216 (7)	0.0196 (7)	0.0205 (7)	0.0085 (6)	0.0037 (6)	0.0050 (6)
C10	0.0305 (8)	0.0269 (8)	0.0195 (7)	0.0110 (6)	0.0033 (6)	0.0056 (6)
C11	0.0374 (8)	0.0340 (9)	0.0196 (7)	0.0155 (7)	0.0096 (6)	0.0117 (7)
C12	0.0304 (8)	0.0297 (8)	0.0291 (8)	0.0120 (6)	0.0140 (6)	0.0150 (7)
C13	0.0232 (7)	0.0207 (7)	0.0260 (8)	0.0084 (6)	0.0099 (6)	0.0097 (6)
C14	0.0244 (7)	0.0214 (7)	0.0289 (8)	0.0068 (6)	0.0099 (6)	0.0075 (6)
C15	0.0289 (8)	0.0244 (8)	0.0252 (8)	0.0063 (6)	0.0072 (6)	0.0054 (6)
C16	0.0280 (8)	0.0256 (8)	0.0278 (8)	0.0040 (6)	0.0015 (6)	0.0092 (6)
C17	0.0194 (7)	0.0183 (7)	0.0392 (9)	0.0025 (6)	−0.0005 (6)	0.0095 (6)
C18	0.0177 (7)	0.0171 (7)	0.0349 (9)	0.0023 (5)	0.0058 (6)	0.0035 (6)
C19	0.0172 (6)	0.0158 (7)	0.0277 (8)	0.0003 (5)	0.0054 (6)	0.0032 (6)
C20	0.0226 (7)	0.0178 (7)	0.0261 (8)	−0.0002 (6)	0.0037 (6)	0.0030 (6)
C21	0.0266 (8)	0.0280 (8)	0.0262 (8)	0.0016 (6)	0.0049 (6)	−0.0002 (6)
C22	0.0377 (9)	0.0377 (10)	0.0247 (8)	−0.0031 (7)	0.0080 (7)	−0.0020 (7)
C23	0.0453 (10)	0.0366 (9)	0.0248 (8)	−0.0027 (8)	0.0023 (7)	0.0079 (7)
C24	0.0391 (9)	0.0321 (9)	0.0316 (9)	0.0070 (7)	0.0026 (7)	0.0131 (7)

### Geometric parameters (Å, °)

**Table:**

Al1—O8	1.9162 (11)	C3—H3	0.9500
Al1—O2	1.9178 (11)	C4—C5	1.391 (2)
Al1—O5	1.9211 (11)	C4—H4	0.9500
Al1—O3	1.9226 (10)	C5—C6	1.382 (2)
Al1—N1	1.9341 (12)	C5—H5	0.9500
Al1—N2	1.9390 (12)	C6—C7	1.516 (2)
O1S—H1B	0.8499	C8—C9	1.516 (2)
O1S—H1A	0.8501	C9—C10	1.381 (2)
O1—C1	1.2153 (18)	C10—C11	1.394 (2)
O2S—H2A	0.8499	C10—H10	0.9500
O2S—H2B	0.8501	C11—C12	1.393 (2)
O2—C1	1.3016 (18)	C11—H11	0.9500
O3S—H3B	0.8502	C12—C13	1.384 (2)
O3S—H3A	0.8499	C12—H12	0.9500
O3—C7	1.2901 (17)	C13—C14	1.518 (2)
O4—C7	1.2245 (17)	C15—C16	1.371 (2)
O5—C8	1.2908 (17)	C15—H15	0.9500
O6—C8	1.2251 (18)	C16—C17	1.385 (2)
O7—C14	1.2265 (18)	C16—H16	0.9500
O8—C14	1.2938 (18)	C17—C18	1.386 (2)
N1—C2	1.3320 (18)	C17—H17	0.9500
N1—C6	1.3364 (17)	C18—C19	1.386 (2)
N2—C9	1.3320 (18)	C18—H18	0.9500
N2—C13	1.3358 (18)	C19—C20	1.473 (2)
N3—C15	1.3373 (19)	C20—C21	1.393 (2)
N3—C19	1.3548 (18)	C21—C22	1.381 (2)
N3—H3C	0.8532	C21—H21	0.9500
N4—C24	1.340 (2)	C22—C23	1.379 (3)
N4—C20	1.3434 (19)	C22—H22	0.9500
C1—C2	1.5157 (19)	C23—C24	1.384 (2)
C2—C3	1.383 (2)	C23—H23	0.9500
C3—C4	1.393 (2)	C24—H24	0.9500
			
O8—Al1—O2	91.91 (5)	O3—C7—C6	113.16 (11)
O8—Al1—O5	159.66 (5)	O6—C8—O5	126.52 (13)
O2—Al1—O5	92.30 (5)	O6—C8—C9	120.22 (13)
O8—Al1—O3	92.25 (5)	O5—C8—C9	113.26 (12)
O2—Al1—O3	159.56 (5)	N2—C9—C10	120.48 (13)
O5—Al1—O3	90.72 (5)	N2—C9—C8	109.84 (12)
O8—Al1—N1	101.41 (5)	C10—C9—C8	129.67 (13)
O2—Al1—N1	79.78 (5)	C9—C10—C11	117.41 (14)
O5—Al1—N1	98.92 (5)	C9—C10—H10	121.3
O3—Al1—N1	79.79 (5)	C11—C10—H10	121.3
O8—Al1—N2	79.79 (5)	C12—C11—C10	121.29 (14)
O2—Al1—N2	99.48 (5)	C12—C11—H11	119.4
O5—Al1—N2	79.89 (5)	C10—C11—H11	119.4
O3—Al1—N2	100.95 (5)	C13—C12—C11	117.84 (14)
N1—Al1—N2	178.59 (5)	C13—C12—H12	121.1
H1B—O1S—H1A	107.3	C11—C12—H12	121.1
H2A—O2S—H2B	99.0	N2—C13—C12	119.83 (14)
C1—O2—Al1	119.04 (9)	N2—C13—C14	109.62 (12)
H3B—O3S—H3A	101.0	C12—C13—C14	130.55 (13)
C7—O3—Al1	118.80 (9)	O7—C14—O8	125.69 (14)
C8—O5—Al1	118.65 (9)	O7—C14—C13	121.32 (13)
C14—O8—Al1	119.08 (9)	O8—C14—C13	112.98 (12)
C2—N1—C6	122.76 (12)	N3—C15—C16	119.62 (14)
C2—N1—Al1	118.66 (9)	N3—C15—H15	120.2
C6—N1—Al1	118.58 (9)	C16—C15—H15	120.2
C9—N2—C13	123.14 (12)	C15—C16—C17	118.74 (14)
C9—N2—Al1	118.34 (9)	C15—C16—H16	120.6
C13—N2—Al1	118.52 (10)	C17—C16—H16	120.6
C15—N3—C19	123.93 (12)	C16—C17—C18	120.49 (14)
C15—N3—H3C	116.3	C16—C17—H17	119.8
C19—N3—H3C	119.6	C18—C17—H17	119.8
C24—N4—C20	116.89 (14)	C17—C18—C19	119.55 (13)
O1—C1—O2	126.75 (13)	C17—C18—H18	120.2
O1—C1—C2	120.76 (13)	C19—C18—H18	120.2
O2—C1—C2	112.49 (12)	N3—C19—C18	117.66 (13)
N1—C2—C3	120.37 (13)	N3—C19—C20	116.26 (12)
N1—C2—C1	110.01 (12)	C18—C19—C20	126.07 (13)
C3—C2—C1	129.62 (13)	N4—C20—C21	123.42 (14)
C2—C3—C4	117.76 (13)	N4—C20—C19	114.72 (13)
C2—C3—H3	121.1	C21—C20—C19	121.85 (14)
C4—C3—H3	121.1	C22—C21—C20	118.29 (15)
C5—C4—C3	120.97 (13)	C22—C21—H21	120.9
C5—C4—H4	119.5	C20—C21—H21	120.9
C3—C4—H4	119.5	C23—C22—C21	119.13 (15)
C6—C5—C4	117.92 (13)	C23—C22—H22	120.4
C6—C5—H5	121.0	C21—C22—H22	120.4
C4—C5—H5	121.0	C22—C23—C24	118.73 (16)
N1—C6—C5	120.20 (13)	C22—C23—H23	120.6
N1—C6—C7	109.62 (12)	C24—C23—H23	120.6
C5—C6—C7	130.17 (12)	N4—C24—C23	123.51 (16)
O4—C7—O3	126.31 (13)	N4—C24—H24	118.2
O4—C7—C6	120.53 (13)	C23—C24—H24	118.2
			
O8—Al1—O2—C1	101.43 (10)	C4—C5—C6—N1	0.6 (2)
O5—Al1—O2—C1	−98.48 (10)	C4—C5—C6—C7	−178.88 (13)
O3—Al1—O2—C1	−0.2 (2)	Al1—O3—C7—O4	−177.70 (11)
N1—Al1—O2—C1	0.17 (10)	Al1—O3—C7—C6	2.38 (15)
N2—Al1—O2—C1	−178.61 (10)	N1—C6—C7—O4	177.78 (12)
O8—Al1—O3—C7	−102.56 (10)	C5—C6—C7—O4	−2.7 (2)
O2—Al1—O3—C7	−0.96 (19)	N1—C6—C7—O3	−2.29 (16)
O5—Al1—O3—C7	97.57 (10)	C5—C6—C7—O3	177.20 (13)
N1—Al1—O3—C7	−1.36 (10)	Al1—O5—C8—O6	179.47 (12)
N2—Al1—O3—C7	177.41 (10)	Al1—O5—C8—C9	−0.78 (15)
O8—Al1—O5—C8	3.61 (19)	C13—N2—C9—C10	0.3 (2)
O2—Al1—O5—C8	−98.17 (10)	Al1—N2—C9—C10	−179.53 (10)
O3—Al1—O5—C8	102.05 (10)	C13—N2—C9—C8	−179.09 (12)
N1—Al1—O5—C8	−178.18 (10)	Al1—N2—C9—C8	1.05 (14)
N2—Al1—O5—C8	1.06 (10)	O6—C8—C9—N2	179.59 (13)
O2—Al1—O8—C14	98.96 (10)	O5—C8—C9—N2	−0.18 (16)
O5—Al1—O8—C14	−2.9 (2)	O6—C8—C9—C10	0.2 (2)
O3—Al1—O8—C14	−101.06 (10)	O5—C8—C9—C10	−179.52 (14)
N1—Al1—O8—C14	178.91 (10)	N2—C9—C10—C11	−0.6 (2)
N2—Al1—O8—C14	−0.33 (10)	C8—C9—C10—C11	178.64 (13)
O8—Al1—N1—C2	−88.99 (10)	C9—C10—C11—C12	0.2 (2)
O2—Al1—N1—C2	0.90 (10)	C10—C11—C12—C13	0.5 (2)
O5—Al1—N1—C2	91.64 (10)	C9—N2—C13—C12	0.5 (2)
O3—Al1—N1—C2	−179.24 (10)	Al1—N2—C13—C12	−179.68 (10)
O8—Al1—N1—C6	90.13 (10)	C9—N2—C13—C14	−179.37 (12)
O2—Al1—N1—C6	−179.98 (10)	Al1—N2—C13—C14	0.49 (14)
O5—Al1—N1—C6	−89.24 (10)	C11—C12—C13—N2	−0.9 (2)
O3—Al1—N1—C6	−0.12 (10)	C11—C12—C13—C14	178.93 (14)
O8—Al1—N2—C9	179.73 (11)	Al1—O8—C14—O7	−179.45 (12)
O2—Al1—N2—C9	89.49 (10)	Al1—O8—C14—C13	0.67 (15)
O5—Al1—N2—C9	−1.17 (10)	N2—C13—C14—O7	179.40 (13)
O3—Al1—N2—C9	−89.94 (10)	C12—C13—C14—O7	−0.4 (2)
O8—Al1—N2—C13	−0.14 (10)	N2—C13—C14—O8	−0.72 (17)
O2—Al1—N2—C13	−90.38 (10)	C12—C13—C14—O8	179.47 (14)
O5—Al1—N2—C13	178.96 (11)	C19—N3—C15—C16	−0.3 (2)
O3—Al1—N2—C13	90.20 (10)	N3—C15—C16—C17	−0.6 (2)
Al1—O2—C1—O1	179.55 (12)	C15—C16—C17—C18	0.8 (2)
Al1—O2—C1—C2	−1.03 (15)	C16—C17—C18—C19	0.0 (2)
C6—N1—C2—C3	−1.4 (2)	C15—N3—C19—C18	1.1 (2)
Al1—N1—C2—C3	177.65 (10)	C15—N3—C19—C20	−178.35 (13)
C6—N1—C2—C1	179.33 (12)	C17—C18—C19—N3	−0.9 (2)
Al1—N1—C2—C1	−1.59 (14)	C17—C18—C19—C20	178.44 (13)
O1—C1—C2—N1	−178.90 (13)	C24—N4—C20—C21	0.2 (2)
O2—C1—C2—N1	1.64 (16)	C24—N4—C20—C19	−178.64 (13)
O1—C1—C2—C3	1.9 (2)	N3—C19—C20—N4	13.58 (18)
O2—C1—C2—C3	−177.51 (13)	C18—C19—C20—N4	−165.77 (13)
N1—C2—C3—C4	0.6 (2)	N3—C19—C20—C21	−165.29 (13)
C1—C2—C3—C4	179.71 (13)	C18—C19—C20—C21	15.4 (2)
C2—C3—C4—C5	0.7 (2)	N4—C20—C21—C22	−1.5 (2)
C3—C4—C5—C6	−1.3 (2)	C19—C20—C21—C22	177.23 (14)
C2—N1—C6—C5	0.82 (19)	C20—C21—C22—C23	1.3 (2)
Al1—N1—C6—C5	−178.27 (10)	C21—C22—C23—C24	0.1 (2)
C2—N1—C6—C7	−179.64 (11)	C20—N4—C24—C23	1.3 (2)
Al1—N1—C6—C7	1.28 (14)	C22—C23—C24—N4	−1.5 (3)

### Hydrogen-bond geometry (Å, °)

**Table:**

*D*—H···*A*	*D*—H	H···*A*	*D*···*A*	*D*—H···*A*
O1S—H1B···O2	0.85	1.98	2.8166 (14)	166
O1S—H1A···O3S^i^	0.85	1.92	2.7472 (18)	165
O2S—H2A···O1S^ii^	0.85	1.92	2.7650 (17)	175
O2S—H2B···O6	0.85	1.92	2.7703 (16)	174
O3S—H3B···O7	0.85	2.02	2.8647 (16)	170
O3S—H3A···O2S^iii^	0.85	1.94	2.7886 (18)	172
N3—H3C···O4^iv^	0.85	2.04	2.7312 (15)	138
N3—H3C···N4	0.85	2.31	2.6497 (19)	104
C12—H12···O1S^i^	0.95	2.46	3.372 (2)	160
C15—H15···O4^iv^	0.95	2.52	2.965 (2)	109
C16—H16···O2S^iii^	0.95	2.33	3.248 (2)	162
C17—H17···O1^v^	0.95	2.25	3.136 (2)	155
C18—H18···O8^vi^	0.95	2.50	3.331 (2)	146
C1—O1···Cg1^vii^	1.2153 (18)	3.4924 (14)	3.9906 (17)	105.38 (10)
C7—O4···Cg2^vi^	1.2245 (17)	3.2311 (13)	3.4319 (17)	88.84 (9)
C1—O1···Cg3^vii^	1.2153 (18)	3.5731 (14)	3.8161 (18)	92.10 (9)

Symmetry codes: (i) −*x*+1, −*y*+1, −*z*; (ii) *x*−1, *y*, *z*; (iii) *x*+1, *y*+1, *z*; (iv) *x*+1, *y*, *z*;  (v) *x*, *y*+1, *z*; (vi) −*x*+1, −*y*+2, −*z*+1; (vii) −*x*+1, −*y*+1, −*z*+1.
